# The Use of Porous Titanium Metal Augments for Acetabular Defects in Total Hip Arthroplasty: Initial Results From a Single-Center Experience

**DOI:** 10.7759/cureus.77307

**Published:** 2025-01-12

**Authors:** Zelimir Jovanovic, Boris Vukomanovic, Dejan Aleksandric, Danilo Jeremic, Lazar Miceta, Nikola D Zarkovic, Nemanja Slavkovic

**Affiliations:** 1 Orthopaedics and Traumatology, Institute for Orthopaedics Banjica, Belgrade, SRB

**Keywords:** acetabulum defects, porous metal augments, titanium implants, total hip arthroplasty, total hip arthroplasty revision

## Abstract

Background and objective

One of the major challenges that orthopaedic surgeons face during revision total hip arthroplasty (THA) is the presence of acetabular bone defects. Nowadays, preference is given to metal augments when it comes to segmental, uncontained defects. This study aimed to present the previous experience at our hospital in the use of porous metal augments for bony defects of the acetabulum.

Methods

Our retrospective observational study included a total of 30 patients who underwent revision or primary THA with the use of porous metal acetabular augmentation between 2021 and 2024. The extent and localization of acetabular bone defects were assessed on preoperative X-ray series and CT scans, confirmed intraoperatively, and classified using the Paprosky classification. The functional status of the patients was evaluated using the Modified Harris Hip Score (mHHS). The complications were defined as adverse outcomes requiring rehospitalization and further treatment. Statistical data analysis was performed using SPSS Statistics version 28.0 (IBM Corp., Armonk, NY).

Results

Surgeries were performed by 14 different orthopaedic surgeons, using a posterolateral hip approach in all cases. Pinnacle Multihole® cementless acetabular shell and Gription® TF augment (DePuy Synthes, Raynham, MA) were implanted in all patients. Revision of the acetabular component was indicated due to mechanical complications in 19 patients (64%), and periprosthetic infection in nine (30%) patients. In two cases (6%) metal augment was used in primary hip arthroplasty. As for acetabular defects, Paprosky type IIb defect was observed in nine cases (30%), type IIc in five cases (16%), type IIIa in 12 (40%), and type IIIb defect in four cases (14%). The analysis of mHHS showed a significant improvement after the operative treatment and the completion of the rehabilitation. Complications of surgical treatment were observed in five patients (16%).

Conclusions

Based on our findings, the use of metal augments is a reliable surgical method that enables the surgeon to deal with various defects of the acetabulum bone mass, ensuring the establishment of favourable biomechanical conditions in the hip joint.

## Introduction

The number of revision total hip arthroplasty (THA) cases is constantly on the rise across orthopedic centers worldwide [1]. One of the key challenges that orthopedic surgeons face during revision THA is the presence of acetabular bone defects [2]. These deficiencies in acetabular bone mass can vary in both location and extent, significantly complicating the revision THA procedure [3,4]. According to data from the German Endoprosthesis Registry, aseptic loosening of the acetabular component is one of the most common indications for revision THA, occurring more than twice as often as the loosening of the femoral stem [5,6].

Effective preoperative preparation is crucial for successful revision THA. Key factors influencing decision-making, aside from the patient's overall health and fitness for surgery, include the classification of the bone defect and the selection of appropriate implant materials and surgical techniques [7]. For classifying bone defects, standard hip X-ray series and CT scans with 3D reconstruction are typically sufficient. However, there is still a need for a universal and precise classification system [6,8]. Several classification systems are in use globally, with the Paprosky and the American Academy of Orthopaedic Surgeons (AAOS) classifications being the most prevalent [9,10].

Given the limitations associated with CT diagnostics, particularly concerning artifacts caused by metal foreign bodies, diagnostics can be enhanced with specific radiographic projections. It is noteworthy that special algorithms for metal artifact reduction (MAR) have been developed to improve the interpretation of CT images [11]. While various techniques exist to address acetabular defects, there is currently a distinct preference for using metal augments, particularly for segmental, uncontained defects that do not compromise the continuity of the pelvic ring [7,12,13]. In this study, we present the results of our institution’s experience with porous metal augments in managing acetabular bone defects, with special emphasis on clinical outcomes and radiographic results.

## Materials and methods

Study design and inclusion and exclusion criteria

This retrospective observational study involved 30 patients who underwent either revision or primary hip arthroplasty utilizing porous metal acetabular augmentation between July 2021 and January 2024. The research adhered to the principles outlined in the Declaration of Helsinki (1964). We collected data on patients' clinical characteristics, operative treatments, and postoperative follow-ups by analyzing the health information system. We employed the ICD-10 classification along with the procedural codebook for THA involving metal augments. The exclusion criteria were as follows: (1) incomplete diagnostic workup, (2) reconstruction of acetabular bone defects with solitary bone grafts, and (3) loss of the patient during the follow-up period.

Data collection

Before surgery, all patients underwent a radiograph of the pelvis in two projections (anteroposterior and oblique Judet view), as well as a multi-slice CT (MSCT) scan. The extent and location of bony defects in the acetabulum were assessed from these images and classified according to the system developed by Paprosky et al. [9].

Patients were admitted to the hospital one day before the planned surgery for comprehensive orthopedic, internist, and anesthesiology preparation. The operative procedure was performed under general endotracheal anesthesia. Blood loss during surgery was managed using a specialized autotransfusion system (Cell Saver). To enhance postoperative pain control, a multimodal analgesic approach was implemented, which included parenteral administration of analgesics, local injection of an analgesic solution before closing the surgical incision, and regional block anesthesia. Rehabilitation commenced on the first postoperative day, with partial weight-bearing allowed on the operated leg for the first three weeks. Subsequently, weight-bearing was gradually increased based on each patient's ability to tolerate it. Antibiotic prophylactic treatment was administered for 24 hours post-surgery, and thromboembolic prophylaxis with low-molecular-weight heparin (LMWH) continued for five weeks thereafter.

Statistical analysis

The postoperative radiological evaluation consisted of obtaining an anteroposterior radiograph of the pelvis immediately after surgery, as well as at three and six months postoperatively, and then at regular one-year intervals afterward. The final images obtained during the follow-up period underwent analysis for osseointegration and the presence of radiolucent lines in the three zones, as described by DeLee and Charnley [14]. These lines were evaluated on the contact surfaces of both the acetabular component-bone and metal augment-bone interfaces. Additionally, any loosening of the acetabular component was noted - defined as a change in the angle of inclination of more than 10°, or a horizontal or vertical displacement exceeding 6 mm after correcting for image magnification. Preoperative and postoperative radiological assessments were carried out by two independent orthopedic surgeons who did not participate in the surgical treatment.

The functional status of patients was evaluated using the Modified Harris Hip Score (mHHS) three months post-surgery, following the completion of the rehabilitation program, and these scores were compared with preoperative values. We also analyzed the occurrence of complications throughout the follow-up period, defined as any adverse events or outcomes that necessitated re-hospitalization or further treatment, whether operative or nonoperative. Statistical analysis of the data was performed using SPSS Statistics version 28.0 (IBM Corp., Armonk, NY), employing descriptive and analytical methods.

## Results

**Figure 1:**
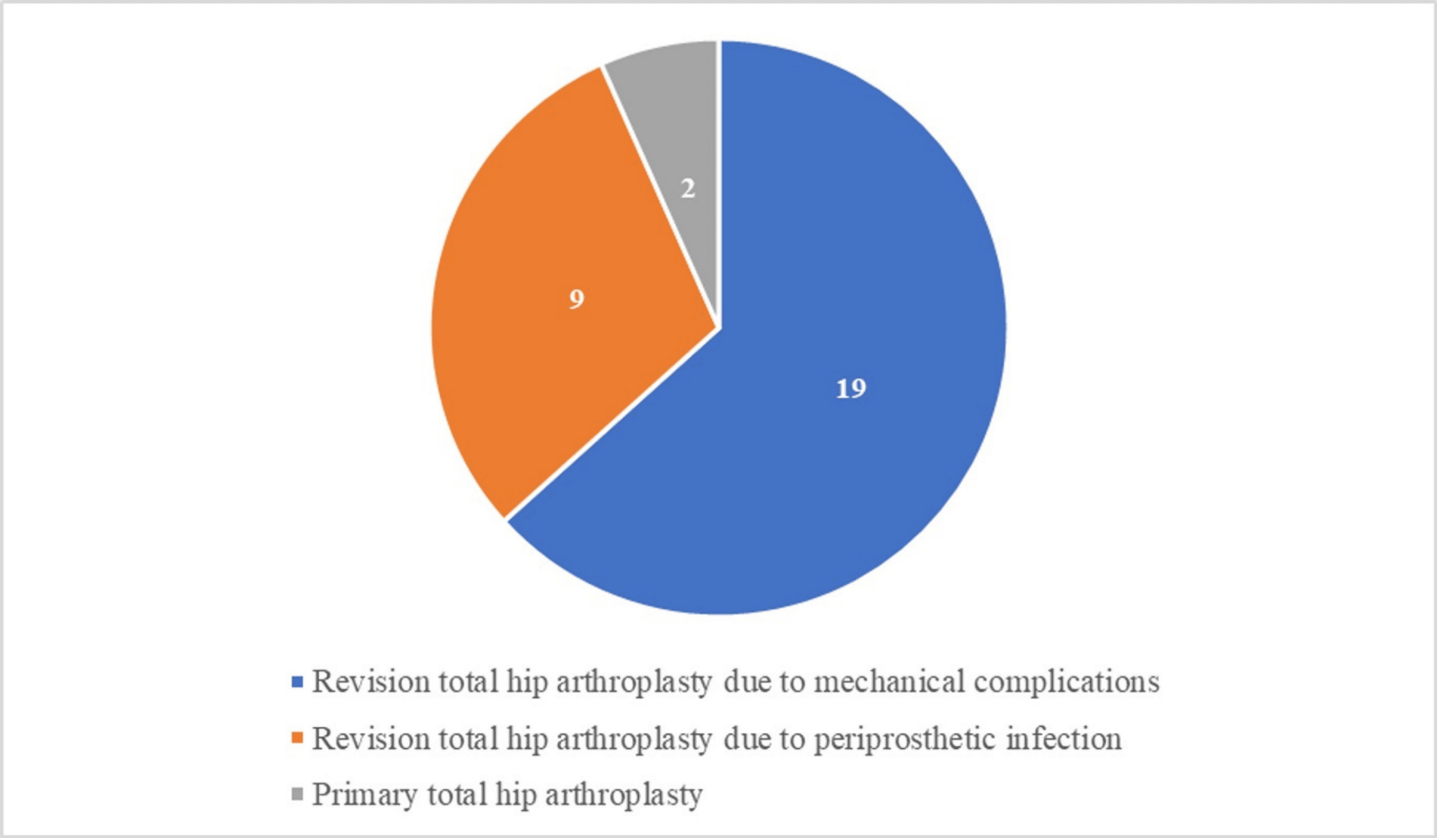
Indications for the use of porous metal augments in our study

In the cohort, 22 (73%) patients were female and eight (27%) were males, with a mean age of 67.9 years at the time of surgery; 28 (94%) underwent revision THA, while two (6%) had primary THA. The indication for primary THA was degenerative hip disease in all patients. Indications for revision THA were mechanical complications (aseptic loosening or recurrent dislocations) in 19 patients (64%), and definitive surgery for periprosthetic infection treatment in nine patients (30%) (Figure 1).

**Figure 2:**
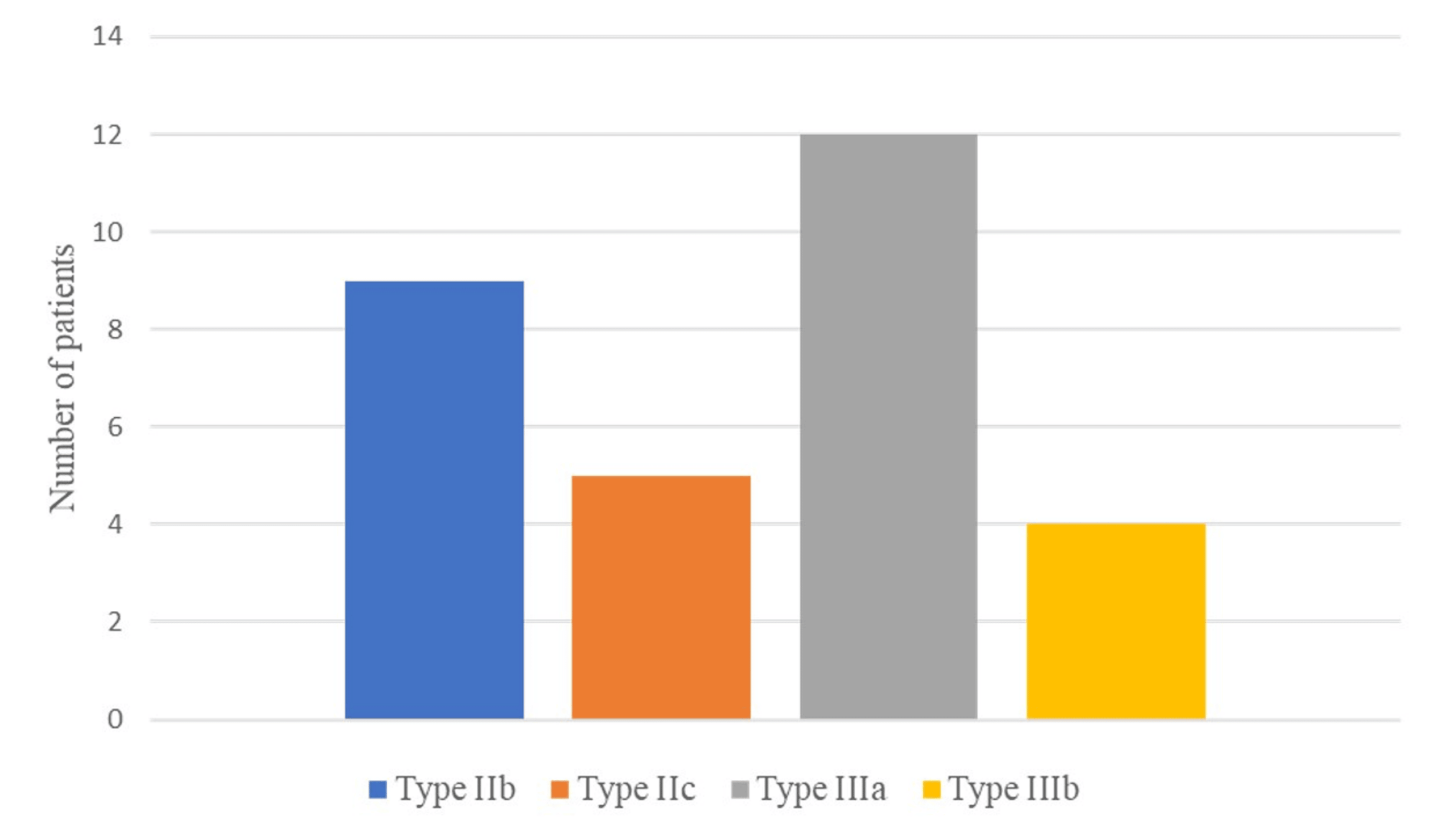
Distribution of acetabular bone defects according to Paprosky classification

The acetabular defect classification was made based on preoperative radiographs and CT imaging; in all cases, it was confirmed by intraoperative findings. Acetabular Paprosky type IIb defect was observed in nine cases (30%), type IIc in five cases (16%), type IIIa in 12 (40%), and type IIIb defect in four cases (14%) (Figure 2).

The surgeries were all performed by 14 different orthopedic surgeons with a minimum of five years of revision THA experience. Both methods of implantation of metal augment (shell-first and augment-first) were used depending on the position of the defect and the preference of the surgeon. The implant of choice (the only one available at our Institution) was a Pinnacle Multihole cementless acetabular shell with Gription TF augment (DePuy Synthes, Raynham, MA). Regardless of the technique used, a layer of Refobacin® cement (Zimmer Biomet, Warsaw, IN) is manually placed on the contact surface between the augment and the acetabular component. 

Postoperative radiographs after three months showed signs of osseointegration of the acetabular component and metal augment in 29 patients (97%). In only one patient (3%), a linear radiolucency was observed in zone 2, with a diameter of less than 2 mm. The patient was symptom-free, and this finding remained unchanged on subsequent X-rays with no signs of loosening.

**Figure 3:**
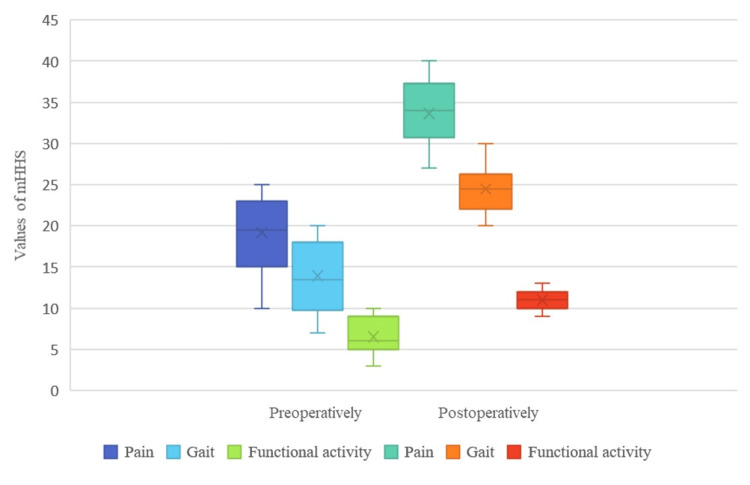
mHHS values preoperatively (left) vs. postoperatively (right) mHHS: Modified Harris Hip Score

The analysis of the value of the mHHS showed a significant improvement after the operative treatment and the completion of the rehabilitation (Figure 3). The mean mHHS value preoperatively was 50.45, which improved to 75.8 postoperatively.

**Figure 4:**
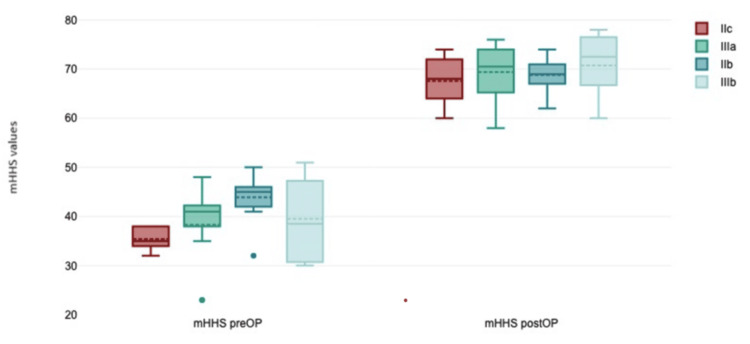
mHHS values according to Paprosky defect type: preoperative (left) vs. postoperative (right) mHHS: Modified Harris Hip Score

We also compared preoperative and postoperative mHHS values for each Paprosky group (IIb, IIc, IIIa, and IIIb) using the one-way ANOVA test. There was a statistically significant improvement in mHHS values after surgical treatment, especially for the IIIb group with the most severe type of acetabular defect (p=0.02) (Figure 4).

The median follow-up time was 18 months (range: 5-36 months). Complications of surgical treatment were observed in five patients (16%). In three patients, dislocation occurred in the early period after the operation. Two of them were successfully treated with closed reduction, while one patient underwent revision surgery due to initially reduced femoral offset. In two patients, an infection of the operative site occurred, which was treated surgically, and in both cases, they were initially treated for periprosthetic joint infection. There were no complications related to bone-implant loosening or construct failure, representing a survival rate of 100% at a mean follow-up of 18 months.

## Discussion

Acetabular bone defects pose significant challenges for orthopedic surgeons in performing successful THA. These defects can result from various processes, including osteolysis, stress shielding, or iatrogenic bone loss during the removal of the acetabular component [7]. Many surgical techniques have been described in the literature for addressing acetabular bone defects [4]. The choice of a particular method depends on several factors, with the most important being the size and location of the defect, the availability of implant materials, and the experience and preferences of specific institutions and surgeons [3].

Historically, cement fixation was the preferred method for revising the acetabular component; however, there has been a recent shift towards cementless acetabular components [15]. While biological fixation has many advantages, achieving adequate initial stability and maximizing the contact surface area to promote better bone ingrowth is a major challenge [16]. A significant advancement in cementless fixation has been the development of extra-large acetabular components, often referred to as jumbo cups, which can effectively bridge acetabular bone defects on their own [15,17]. If this is not feasible, it is recommended to shift the center of rotation proximally to ensure adequate contact with the acetabular walls [18]. However, this approach may complicate stability and the restoration of limb length if a femoral component revision is not performed simultaneously.

Several surgical techniques can be employed when the bone defect is more oval-shaped than hemispherical. Some manufacturers have developed bilobed acetabular components to address these defects [19]. However, a major drawback of these implants is the frequent mismatch between the defect shape and the component, necessitating the removal of additional acetabular bone for a proper fit. A study by Chen et al. reported disappointing outcomes with this method, highlighting a failure rate of 26% over a five-year follow-up period [20].

Sporer et al. described a technique for replacing segmental acetabular defects using a specially designed structural bone allograft shaped like an inverted number seven, derived from the distal femur allograft [21]. This technique enjoyed considerable popularity in the past, with some studies reporting success rates between 80% and 86% [22,23]. However, more recent studies have not replicated these positive results, particularly when the contact area between the acetabular bone and the implant is less than 50% of its surface area. Chandler et al. conducted a study over 12 years and indicated a revision rate of 26%, with 41% of patients showing radiographic signs of aseptic loosening [24]. Additionally, the use of bone allografts carries inherent risks associated with foreign tissue transplantation.

**Figure 5:**
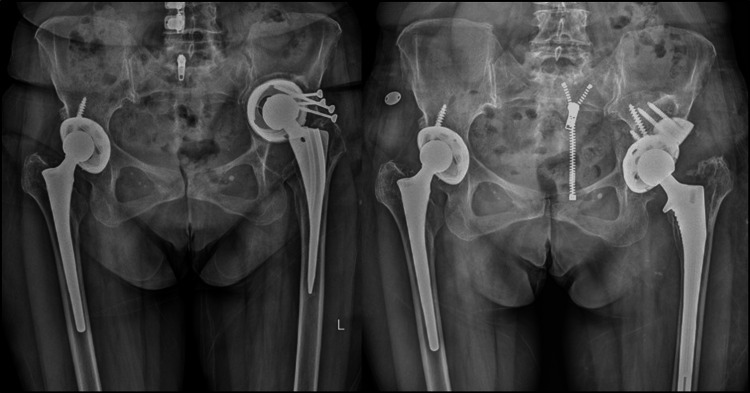
Revision THA with the use of metal augment in 62-year-old female patient indicated for proximal and medial migration of acetabular component (Paprosky defect type IIIa) X-ray before surgery (left); X-ray one year after surgery (right) showing adequate osseointegration of acetabular component and augment THA: total hip arthroplasty Image credits: Zelimir Jovanovic (author)

Metal augments have emerged as a promising alternative, offering numerous advantages over structural bone grafts [25]. These augments, made from trabecular metal (tantalum), closely resemble the structure of cancellous bone and facilitate reliable osseointegration [26]. They are biologically stable, reducing the risk of loosening due to bone graft resorption (Figure 5). Moreover, modular augments come in various sizes and shapes, enabling surgeons to select the option that best matches the bone defect and ensures close contact with the bone tissue [16,25].

Siegmeth et al. conducted a study involving 34 patients who underwent THA using trabecular augments over two years, reporting a negative outcome in only three cases that required revision surgery due to recurrent dislocations [27]. Their findings indicated that metal augments could help establish a normal center of rotation in the hip. Nehme et al. observed two revision cases among 16 patients during a three-year follow-up, none of which were related to the loosening of the acetabular component [16]. In contrast, a larger study conducted by Mahmoud et al. involving 147 patients revealed a complication rate of 9.6%, with revision of the acetabular component required in only half of those cases [28].

The Gription TF® metal augments used in our patients differ in structure and composition from trabecular metal. These augments are constructed from titanium, which has a porosity of 60%, compared to the 80% porosity seen in tantalum augments. Despite these differences in structure and the technological processes involved in their production, both types exhibit similar tribological profiles in terms of friction coefficient and modulus of elasticity [26]. However, Beckmann et al. identified distinct differences in the behavior of these materials under varying load conditions [29]. Their experimental model demonstrated that at lower loads, titanium exhibited less micromotion at the bone-augment interface compared to tantalum, whereas the opposite was true under maximum loads. Although Mahmoud et al. included both types of metal augments in their study, they did not investigate potential differences in clinical outcomes between the two groups [28]. While the proportion of complications in our sample was relatively high, these were not due to implant-bone construct failure or loosening, and we had a 100% survivorship over an average follow-up period of 18 months. Hence, it is reasonable to assume that the differences in these implants are not the primary factors behind surgery failures.

To the best of our knowledge, this study involved the largest reported sample concerning the short-term clinical and radiological behavior of titanium metal augments. Our results align with a large meta-analysis conducted by Banerjee et al., which included over 2,000 patients and found a complication rate of 8.9%, a postoperative Harris Hip Score (HHS) increase of 79 points, and a prevalence of periacetabular radiolucencies at 4.9% [30]. Although we observed a higher complication rate of 16% in our study, no reoperations were required due to the loosening of the acetabular component. The elevated complication rate in our study may be attributed to the fact that metal augments were not used at our institution before the study period, and a diverse group of surgeons, each performing a relatively low number of cases, conducted the procedures. The shallow learning curve associated with a limited number of cases suggests that this surgery should ideally be performed by highly skilled surgeons in centers capable of handling larger volumes.

Finally, it is important to acknowledge several limitations in our study. One significant limitation is the small sample size, which affects the reliability of our results. Additionally, the relatively short follow-up period reduces our ability to draw conclusions about long-term outcomes, particularly the survival of the acetabular component-augment construct. Lastly, the involvement of several different surgeons in the procedures may lead to variations in surgical techniques, potentially influencing the clinical outcomes.

## Conclusions

The use of titanium metal augments is a reliable surgical method for addressing various defects in the acetabulum bone mass. This technique ensures the proper positioning of the acetabular component and promotes favorable biomechanical conditions in the hip joint. Current results from studies utilizing acetabular augments to address acetabular bone defects in THA are promising, with this study confirming excellent short-term survival rates for titanium alloy augment constructs. Given the increasing number of revision THAs, further research is essential to compare the long-term clinical and radiological outcomes of different techniques for managing acetabular bone defects.
